# Predicting new indications of compounds with a network pharmacology approach: Liuwei Dihuang Wan as a case study

**DOI:** 10.18632/oncotarget.21398

**Published:** 2017-09-30

**Authors:** Yin-Ying Wang, Hong Bai, Run-Zhi Zhang, Hong Yan, Kang Ning, Xing-Ming Zhao

**Affiliations:** ^1^ Institute of Science and Technology for Brain-Inspired Intelligence (ISTBI), Fudan University, Shanghai 200433, China; ^2^ Department of Computer Science and Technology, Tongji University, Shanghai 201804, China; ^3^ Department of Electronic Engineering, City University of Hong Kong, Kowloon 999077, Hong Kong; ^4^ Key Laboratory of Molecular Biophysics of the Ministry of Education, College of Life Science and Technology, Huazhong University of Science and Technology, Wuhan, Hubei 430074, China

**Keywords:** network pharmacology, drug repurposing, TCMs, pathway profile, LDW

## Abstract

With the ever increasing cost and time required for drug development, new strategies for drug development are highly demanded, whereas repurposing old drugs has attracted much attention in drug discovery. In this paper, we introduce a new network pharmacology approach, namely PINA, to predict potential novel indications of old drugs based on the molecular networks affected by drugs and associated with diseases. Benchmark results on FDA approved drugs have shown the superiority of PINA over traditional computational approaches in identifying new indications of old drugs. We further extend PINA to predict the novel indications of Traditional Chinese Medicines (TCMs) with Liuwei Dihuang Wan (LDW) as a case study. The predicted indications, including immune system disorders and tumor, are validated by expert knowledge and evidences from literature, demonstrating the effectiveness of our proposed computational approach.

## INTRODUCTION

With the ever increasing cost and time-consuming process of drug development, new strategies for drug development are highly demanded. Drug repurposing, which aims for identifying novel indications for existing drugs, attracts a lot of attention since the toxicity of known drugs is already understood [1]. For example, Metformin has been widely used for more than 30 years for the treatment of type 2 diabetes, but extensive preclinical and clinical studies over the past decade have demonstrated the antitumor effects of the drug [2]. It has been reported that Metformin was able to lower the risk of cancer mortality and incidence in patients with diabetes [3]. Nowadays, drug repurposing has been considered as an effective approach in drug development. However, identifying novel indications with drug repurposing is highly challenging since the novel indications of one drug may have no obvious relationship with its initial purpose.

During the past decade, much effort has been made to develop new computational approaches for the purpose of repositioning drugs and elucidating the molecular mechanisms of drugs. For example, Wang *et al* proposed a novel method to predict drug target proteins based on drug-domain interactions [4], and Zhang *et al* constructed a post-translational regulatory network to explore network motifs as potential drug targets which can help design multi-component or combinatorial drugs [5]. With the popular deep learning (DL) techniques, Kadurin *et al* proposed a DL-based model for screening potential anti-cancer compounds [6]. Recently, the network pharmacology approaches have been widely employed for understanding the mechanisms of drug actions, resistance and side effects [7–9]. At the same time, some network pharmacology approaches have been proposed to predict the associations between drugs and diseases. For instance, Martinez *et al* developed DrugNet to prioritize drugs for certain diseases by integrating complex associations among disease, drugs and proteins [10]. Besides, Alaimo *et al* also introduced a method that can be used to integrate biological knowledge and bipartite interaction network to predict new indications of drugs [11].

As multi-target or multi-component therapies gain increasing attention recently, Traditional Chinese Medicines (TCMs) are being re-evaluated and becoming important resources for the discovery of alternative treatments for certain diseases, where various network pharmacology approaches have been proposed for this purpose [12–15]. For example, Qing Luo Yin (QLY) is an effective formula in the treatment of arthritis and antiangiogenic. With the target network of QLY, not only the diseases related key biological processes including angiogenesis, inflammatory and immune response were revealed, but also the active ingredients and synergistic combinations of this herbal formula were identified [16]. Another example is Liuwei Dihuang Wan (LDW), which shows potential for regulating the imbalance of hormones and metabolism [17]. Therefore, the network pharmacology approaches are capable of providing insights into the mechanisms of actions of known drugs and identifying new indications of those drugs [18–20]. However, current network pharmacology methods for repurposing drugs are mainly based on the target proteins of active compounds, whereas the target information may not be indicative of diseases that the drugs can be used for.

**Figure 1:**
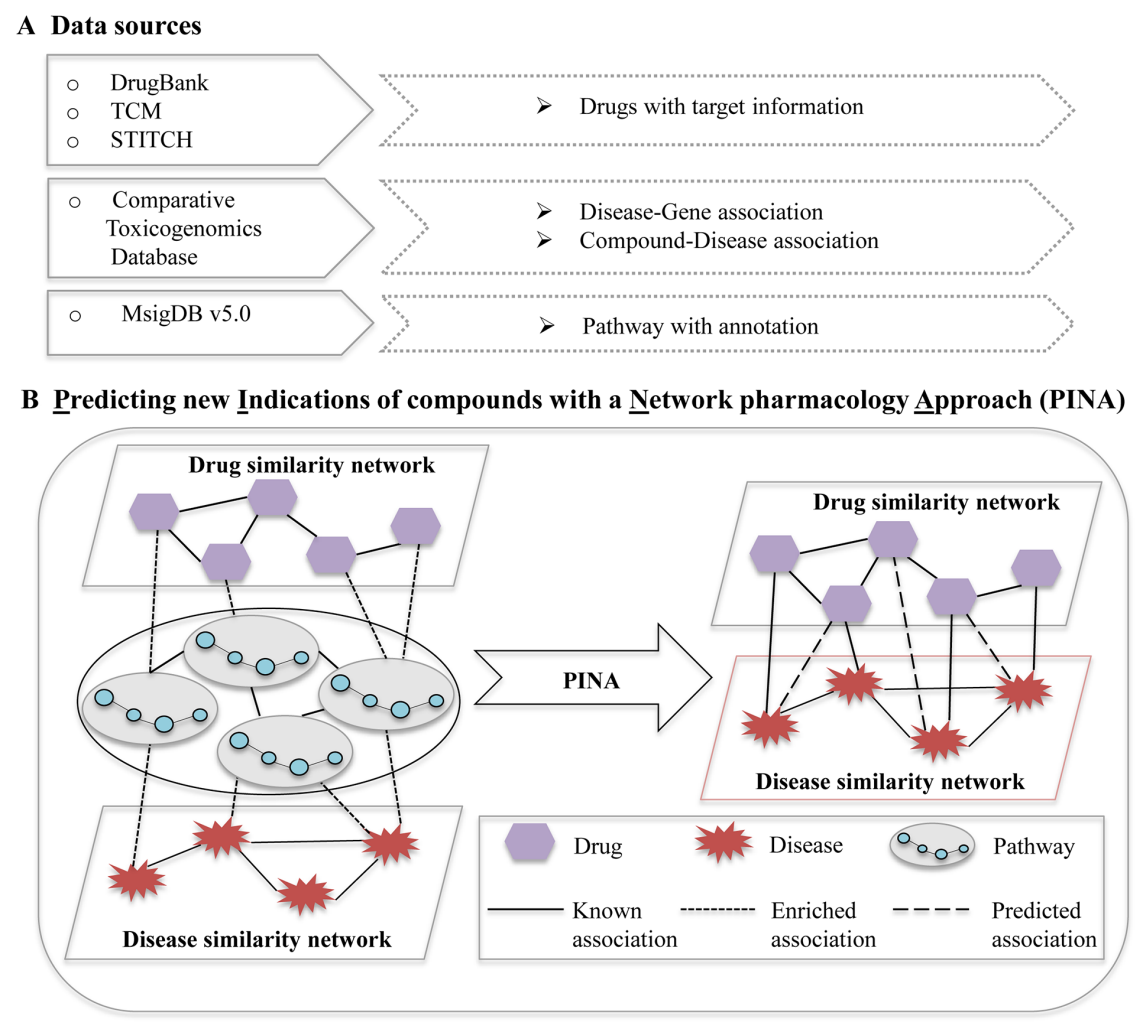
The pipeline of predicting new indications of compounds with a network pharmacology approach **(A)** Data sources for network pharmacology analysis; **(B)** Predicting new indications of compounds with a network pharmacology approach. Node: irregular, disease; hexagon, compound; circle, gene. Line: solid line, known association; square dot, enriched association; long dash, predicted association.

In this paper, we investigate the mechanisms of drug actions based on the pathways modulated by the drugs. By further integrating pathway profiles with chemical structures as well as disease phenotypes, we present a network pharmacology approach namely PINA (Predicting new Indications of compounds with a Network pharmacology Approach) as shown in Figure 1, to predict potential indications of old drugs. Benchmark results on FDA approved drugs have proven the superiority of our method over traditional network pharmacology approaches, as regard to revealing new associations between compounds and diseases. We further extend PINA to predict the novel indications of Traditional Chinese Medicines (TCMs) with Liuwei Dihuang Wan (LDW) as a case study. The predicted indications, including immune system disorders and tumor, are validated by expert knowledge and evidences from literature, demonstrating the effectiveness of our proposed computational approach.

## RESULTS

### Identification of the pathway profiles associated with diseases

In this work, given a disease, we assume that the compounds that can significantly affect the pathway profiles associated with the disease can be used for the disease. With 4,774 known drug-disease associations composed of 928 compounds and 608 diseases extracted from CTD database [21], we first identified the pathways that are dysfunctional in diseases. Assuming that diseases with similar pathway profiles should have similar mechanisms, based on the pathways we identified a disease-disease association network was constructed, where two diseases were linked if they shared at least one pathway. We further detected modules from the network with density-based MCODE [22] tool (Supplementary Figure 2). Table 1 listed the 14 modules and the corresponding average similarities among diseases within the module as well as the most enriched disease class. Supplementary Table 2 has shown the detailed information of the 14 modules. If the pathway profiles we identified are indeed associated with diseases, we expected that the diseases belonging to the same module should have similar mechanisms. It could be seen that the diseases grouped into the same module based on pathway profiles tended to have similar symptoms, where the disease similarity was calculated as described in [23]. Furthermore, the diseases can be grouped into 22 classes based on the physiological systems affected by the diseases as defined in [24]. By investigating the diseases belonging to same module, we found that the diseases in the same module tend to be in the same class as shown in Table 1, indicating that the diseases from the same module have similar mechanisms. In addition, by investigating the number of disease classes that the pathway profiles were associated with, we found that each of more than 79% pathways was associated with only one specific disease class, implying that each pathway profile is specifically associated with a certain type of diseases (Supplementary Figure 3).

**Table 1:** The modules detected by MCODE from the disease association network generated with disease related pathway profiles

Modules	Number of diseases	Average similarity^#^	Disease class (Coverage)^*^
1	16	0.1623	Psychiatric (0.625)
2	9	0.3210	Neurological (0.67)
3	7	0.2448	Ophthamological (0.71)
4	5	0.3294	Connective tissue (0.6)
5	5	0.6367	Cardiovascular (1.0)
6	4	0.4166	Endocrine (1.0)
7	4	0.2861	Neurological (0.5), Metabolic (0.5)
8	4	0.2427	Neurological (0.5), Cancer (0.5)
9	3	0.5193	Neurological (1.0)
10	3	0.3635	Bone (1.0)
11	3	0.2281	Immunological (1.0)
12	3	0.4612	Metabolic (1.0)
13	3	0.4284	Multiple (1.0)
14	3	0.5407	Gastrointestinal (1.0)

^#^Average similarity means the average of similarities over all disease pairs in each module detected in disease associated network.

^*^Coverage means the number of the diseases in the most enriched class divided by the number of disease in the module.

By further investigating the pathway profiles that were associated with one disease class, we found that those pathways were indeed related to the disease class. For instance, the calcium signaling pathway played a crucial role in the control of neuronal functions and plasticity by regulating members of the neuronal calcium sensor (NCS) proteins [25]. It was reported that the deregulation of calcium signaling pathway was one of the key processes in the pathogenesis of neurodegenerative disorders [26]. In our study, the neurological class consists of 48 diseases while the calcium signaling pathway was predicted to be related with 18 out of them. Besides, the transmission across chemical synapses pathway we identified was related to more than 20% of neurological diseases, where the chemical synapses were specialized junctions used for communications between neuron [27]. Furthermore, the GPCR ligand binding pathway was predicted to be associated with all psychiatric diseases, where the G protein-coupled receptors have been found to play important roles in major psychiatric disorders, such as depression and schizophrenia [28].

From the findings shown above, we can see that the pathway profiles identified here are indeed related to the corresponding diseases.

### Prediction of potential indications for FDA approved drugs

With the pathway profiles identified above, the potential associations between compounds and diseases could be predicted. Based on the FDA approved drugs with target information and their known associations with diseases obtained from the CTD database, PIPP, NP__C_ and NP__D_ were respectively applied to predict potential compound-disease associations.

By comparing the three approaches, we noticed that many of the predictions by pathway profile approach (PIPP) could be validated with those predicted by chemical structures and disease similarities based on the ‘guilt by association’ rule, where drugs with similar structures were assumed to be able to treat the same disease while similar diseases could be treated with the same drug. For example, the compound Nortriptyline (CID: 4543) was originally used as an anti-depressive agent [2], and it was predicted for the treatment of schizophrenia (OMIM: 603176) with a score of 0.9985 by PIPP. In fact, the drug Nortriptyline had similar structure with Amitriptyline (CID: 2160), which was used for schizophrenia [9], with a similarity score of 0.92. On the other hand, schizophrenia was similar with Attention Deficit Hyperactivity (ADH) disorder (OMIM: 143465), and Nortriptyline have already been reported for treating ADH in the CTD database, which validated that Nortriptyline could be used for schizophrenia. Moreover, we noticed that the pathway profile approach could successfully recover known associations that were missed by the chemical or disease similarity based approach. For example, the compound Retinoic Acid was used for femur head necrosis, which was successfully identified by our pathway profile method with a score of 1.0. However, the nearest profile approach based on chemical and disease similarity failed to identify this association with scores of 0.0 and 0.27, respectively.

The results shown above demonstrate that the pathway profile approach can complement with other approaches, e.g. chemical or disease similarity based ones, very well. Therefore, we further proposed an ensemble approach named as PINA that combines the pathway profile method with chemical and disease similarity based methods to predict potential compound-disease associations. The novel potential indications of all compounds are list in Supplementary Table 4. We also compared PINA with three existing methods from literature, including DrugNet [10], HGBI [29] and NBI [30]. DrugNet is a network-based drug repositioning method, which integrates the information of diseases, drugs and proteins to prioritize drug-disease associations. HGBI and NBI have been originally developed for predicting drug-protein interactions, and can also be used for the prediction of drug-disease associations. HGBI predicts the drug-disease associations with the guilt-by-association principle based on the drug-disease heterogeneous graph, while NBI can predict new drug-disease associations based on a two-step diffusion model on a drug-disease bipartite graph. To evaluate the performance of our approach, PINA was compared with the other three approaches on the same benchmark drug-disease associations from the Comparative Toxicogenomics Database, where the same pre-process was used for all the four computational approaches. The chemical similarities between compounds were calculated based on their fingerprints by using the Single Linkage algorithm [31] while the disease similarities were defined as described in [23]. All the four approaches were evaluated with 5-fold cross-validations. Table 2 shows the performances of different methods, from which we can see that PINA has the highest AUC (0.8969) and F1 (0.3833) and significantly outperforms the other approaches.

**Table 2:** The performances of different methods which were obtained with 5-fold cross-validation

Method	AUC	Precision	Recall	F1 score
*PIPP*	0.8515	0.1517	0.4899	0.2313
*NP*_*_C*_	0.8132	0.0873	**0.6511**	0.1539
*NP*_*_D*_	0.8633	0.3005	0.4760	0.3684
*PINA*	**0.8969**	**0.4325**	0.3446	**0.3833**
*DrugNet*	0.8034	0.3411	0.3923	0.3568
*HGBI*	0.8125	0.3867	0.3639	0.3752
*NBI*	0.7983	0.3297	0.3321	0.3308

AUC - Area under ROC curve;

Precision - TP/(TP+FP), positive predictive value;

Recall - TP/(TP+FN), true positive rate;

F1 score - Harmonic mean of precision and recall.

### Prediction of potential indications for LDW

In this part, we further extended PINA to predict the novel indications of Traditional Chinese Medicines (TCMs) with Liuwei Dihuang Wan (LDW) as a case study. With the known compound-disease associations from the CTD database, we built a model as described in Equation (4) and identified 59 diseases that LDW can be used for. Among the 156 compound components of LDW, only the eight compounds that can be found new indications with PINA were considered here, where the eight compounds were further required to be drug-like. Table 3 shows the detailed information about the eight compounds. By investigating the indications of the eight compounds obtained from CTD, we found that LDW, as a mixture of multiple compounds, achieves its therapeutic effects through its individual components. For example, LDW was used for anti-aging, delayed development and blurred vision, whereas Retinol, also known as vitamin A, plays an essential role in anti-aging, promoting bone growth and the treatment of various eye conditions. Moreover, it was found that LDW was useful for decreasing blood sugar, suppressing blood pressure and improving the renal function. Another compound component Quercetin, an antioxidant, was reported to treat many LDW associated disease, such as acute kidney injury, diabetes mellitus and hypertension. The combination of Nicotinamide and Retinol could be effective for acne treatment for which LDW has been used for [32].

**Table 3:** The detail information about eight compounds belonging to LDW

Compound ID	Name	FDA Status	Part of known indications obtained from CTD database
CID000445354	Retinol	Approved	Acne Vulgaris; Acute Kidney Injury; Adrenal Insufficiency; Carcinoma, Hepatocellular; Colonic Neoplasms; Diabetes Mellitus, Type 1; Fatty Liver; Hypertension, Portal; Hypertriglyceridemia; Liver Cirrhosis; Nephrosis;
CID000024360	Camptothecin	Experimental	Neoplasms; Leukemia, Lymphoid
CID027237R1936	Nicotinamide	Experimental	Diabetes Mellitus, Type 2; Hypercholesterolemia; Hyperglycemia; Hyperlipoproteinemias; Hypertension; Hypertriglyceridemia; Kidney Diseases; Nerve Degeneration; Ventricular Dysfunction, Left;
CID000006137	L-methionine	Approved	Carcinoma, Hepatocellular; Fatty Liver; Kidney Diseases; Memory Disorders;
CID005280343	Quercetin	Experimental	Acute Kidney Injury; Autoimmune Diseases; Breast Neoplasms; Cognition Disorders; Diabetes Mellitus; Fatty Liver; Hypertension; Kidney Diseases; Memory Disorders; Ovarian Neoplasms; Prostatic Neoplasms;
CID000005641	Urethane	Withdrawn	Arrhythmias, Cardiac; Hypertension; Liver Neoplasms; Ovarian Neoplasms;
CID027237R1305	Choline	Approved	Amnesia; Cognition Disorders; Fatty Liver; Memory Disorders;
CID027237R1681	Dopamine	Approved	Acute Kidney Injury; Arrhythmias, Cardiac; Central Nervous System Diseases; Heart Failure; Hypertension; Learning Disorders; Nerve Degeneration; Nervous System Diseases; Parkinson Disease;

With the findings above, we assumed that the new indications we predicted for LDW can be validated with the indications of its component compounds. Figure 2 shows a compound-disease association network constructed with the associations between diseases and compounds we have predicted, where the 59 diseases that LDW has been predicted to be used for were linked to the eight individual compounds based on PINA. Among the 104 compound-disease associations shown in Figure 2, 21 of them have been reported in CTD. For example, Quercetin could inhibit the growth of MCF-7 breast cancer cell line and promoted apoptosis by reducing G0/G1 phase arrest [33, 34]. Besides, it was widely accepted that the compound could be used to treat a certain disease if it targeted the disease related genes. With the known target and disease genes information, we also found there are another 27 predicted compound-disease associations which can be validated by sharing same genes. For instance, dopamine D2 receptor (DRD2) played an essential role in dopamine signaling which was strongly implicated in the etiology of schizophrenia (SZ) [35], and was also one of the targets of Dopamine. By targeting the gene DRD2, Dopamine may be used for schizophrenia. As a result, 48 compound-disease associations can be validated by difference evidence while the rest associations need further experimental validation.

**Figure 2:**
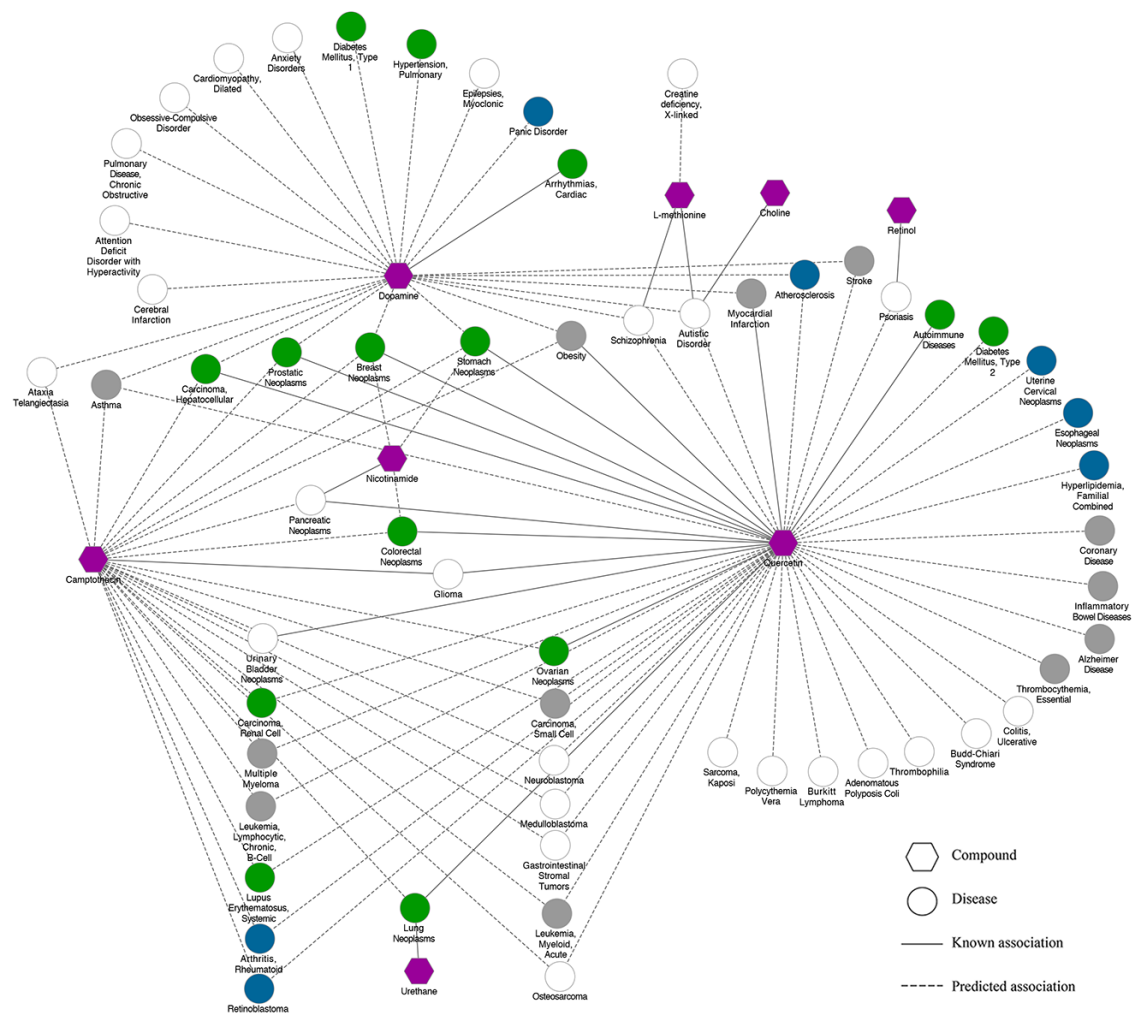
The LDW associated compound-disease network Node: green circles, diseases for which LDW was known to be used for (Number=14); blue circles, diseases reported in Li *et al* that LDW can be used for (Number=7); grey circles, diseases reported in literature that LDW can be used for (Number=12); white circles, diseases predicted to be treated with LDW (Number=26); purple hexagons, compounds. Line: solid lines, known associations between compounds and diseases; dotted lines, predicted associations between compounds and diseases.

By considering the indications (59 diseases) we predicted for LDW, we further investigated whether LDW has been reported to be effective for some of these diseases in literature by expert knowledge. As a result, 14 of them have been known to be associated with LDW as shown in Supplementary Table 3. For example, it was found that LDW could significantly inhibit the breast cancer tumor growth and progression, and promoted the recovery of breast ducts in mice [36, 37]. Likewise, LDW decoction could exert therapeutic effects on liver cancer in mice by affecting tumor cell cycle and down-regulate serum VEGF level [38]. Moreover, it was well accepted that LDW could counteract the adverse effect of steroid and immunosuppressive agents, significantly improving the therapeutic effectiveness in the treatment of Systemic Lupus Erythematosus (SLE) [39]. Besides, many other predicted diseases, i.e. diabetes mellitus, hypertension and so on [40–43], were also known to be associated with LDW.

Previously, Li *et al* predicted new indications for LDW based on drug targets and disease genes [17]. We further investigated how many of our predictions could be validated by those reported in their work, and the new indications found by both works for LDW would be more convincing. Consequently, 7 of our predictions were also reported by Li *et al,* including atherosclerosis, retinoblastoma, rheumatoid arthritis, esophageal neoplasms, uterine cervical neoplasms, familial combined hyperlipidemia and panic disorder. For example, it was found that LDW had already been reported for treating esophageal neoplasms [44]. In addition, studies have shown that LDW pills could effectively inhibit the expression of IL-beta, MMP-1 and MMP-3, thus protecting and repairing the articular cartilage which had significant therapeutic effects on Osteoarthritis [45].

Moreover, we also performed text mining by querying the PubMed database to see whether LDW have been reported effective for the rest of our predictions. As a result, except the diseases mentioned above, 12 diseases have been reported to be treated by LDW in literature as listed in Supplementary Table 3. For instance, it was found that LDW could simultaneously disturb the regulations of apoptosis and protein ubiquitination among biological processes, such as RPS6KA1, FHIT and AMFR, which may be the therapeutic targets of Alzheimer Disease [46, 47]. Moreover, traditional Chinese doctors have already used LDW to treat asthma patients based on the cytokine gene expression perturbed by LDW [48].

Taken together, 33 out of 59 diseases we predicted to be treated by LDW have been validated in different ways, where the known indications with direct evidences tend to rank top. These results demonstrate that LDW can really work for those diseases. The detailed results with corresponding evidences were presented in Supplementary Table 3.

## DISCUSSION

Repurposing old drugs has drawn increasing attention, since they could serve as the effective and cost-saving strategies for drug discovery. In this study, we first introduce pathway profiles associated with diseases and affected by compounds. By integrating the pathway profiles with chemical structure as well as disease phenotype, we present PINA to predict new indications of compounds. Benchmark results on FDA approved drugs have demonstrated the predictive power of PINA. We further extended PINA to predict the potential indications of traditional Chinese medicine with LDW as a case study. The new indications we predicted for LDW have been validated with expert knowledge and evidences from literature.

We also noticed that improvement of our PINA approach is possible when predicting novel indications of TCMs. For example, a TCM formula is typically composed of multiple herbs or hundreds of chemical compounds. Here, the indications of a TCM formula were predicted with a Bayesian model, where the compound components were regarded to be independent with each other. Although the synergistic effects among compounds cannot be explicitly described in the Bayesian model, the good performance on LDW shows the effectiveness of the model. In the future, more efficient models should be developed to take into account the synergistic effects among compounds. Another concern is that many compound components of TCMs are not known while it is expensive and time-consuming to determine all bioactive compounds of TCMs, a comprehensive knowledgebase about compound components of TCMs is highly demanded.

## MATERIALS AND METHODS

### Data sources

The FDA approved human drugs used in our study were retrieved from the DrugBank database (Version 4.3) [49], of which we only focused on the 932 compounds that had target information according to the DrugBank and STITCH databases (Version 4) [50] which provides a confidence score for each interaction. Here, a score of 700 was used as threshold to choose the high-confidence interactions [51]. Specifically, the interactions marked with prediction or text mining were removed to make sure high-quality interactions used in this paper. The LDW was composed of *Rehmannia glutinosa Libosch.*, *Cornus officinalis Sieb. et Zucc., Paeonia suffruticosa Andr., Dioscorea opposita Thunb., Poria cocos (Schw.) Wolf and Alisma orientalis (Sam.) Juzep.* In our work, the chemical constituents of LDW were mainly obtained from the TCM Database@Taiwan [52] by searching the herb names. Meanwhile, the other constituents were also collected manually from published articles by text mining. Then we transformed all constituents into mol2 format with ChemDraw software (http://www.cambridgesoft.com/software/ChemDraw/), and the chemicals were then converted into the canonical SMILES format. We downloaded all known chemicals with each of them annotated with PubChem identity from STITCH database (version 4.0). By querying the known compounds with the chemical SMILES files, the chemical constituents of LDW can be identified. Here, we only picked up the chemicals that had target information according to DrugBank and STITCH databases. Consequently, 156 compounds of LDW (Supplementary Table 1) were finally collected.

The disease-gene associations were obtained from the Comparative Toxicogenomics Database (CTD) [21]. As a result, the associations between 4937 diseases and 8536 genes were collected. We further collected compound-disease associations from the CTD database, and the 4774 associations with direct evidence (therapeutic/maker) between 928 compounds and 608 diseases were used as the positive set while the other possible compound-disease associations were used as the negative set.

All predefined biological pathways used in this study were obtained from the Molecular Signatures Database (Version 5.0) [17], where the canonical pathways from the curated (c2) gene sets were adopted. The physical protein-protein interactions were obtained from HPRD [53], BioGRID [54], IntAct [55], MINT [56] and DIP [57] databases.

### Predicting new indications of compounds with a network pharmacology approach

#### Predicting indications of compounds based on pathway profile

We assumed that the occurrence of a disease was due to the aberrant functions of certain pathways. Accordingly, to treat a disease, the drugs should affect the dysfunctional pathways that were associated with the disease. With this assumption, for each drug and its related disease, the pathways linking the pair of drug and disease were firstly identified. For this, the pathway profiles associated with a drug and a disease were respectively identified, where the drug related pathways were enriched by its target proteins while the disease associated pathways were enriched by its related genes [58]. Given a pair of pathways respectively associated with a drug and a disease, we only considered the pathways that met one of the following conditions: (1) the two pathways are the same one (common pathway); (2) the two pathways share at least one gene (cross-talking pathways); (3) there are protein interactions between the two pathways (interacting pathways) (As shown in Supplementary Figure 1). To avoid possible false positives, the cross-talking or interacting pathways were required to have correlated activities based on the gene expression data obtained from 36 normal tissues [59]. Here, the pathway activity in a tissue was defined as the average expression value of all genes within the pathway and only the pathway pairs with a significant correlation coefficient (p-value <0.01) in 36 tissues were kept for further analysis.

Here, the pathway profile method named PIPP (predicting indications based on pathways profile) were proposed. Given one disease and related drugs as well as the pathways associated with any pair of drug and disease as defined above, the score of a pathway pair associating a drug with the disease it could be used for was defined as follows:

$$P\left(p_{i}\_D\right)=\frac{N\left(C|p_{i}\right)}{N\left(C^{\prime }|D\right)}$$ (1)

where *N*(*C* | *p*_*i*_) is the number of compounds treating disease *D* and the pathway pair *p*_*i*_ is the one that occurs commonly between compound set C and the disease D, and *N*(*C*′ | *D*) is the number of all compounds used for disease *D*. If *P*(*p*_*i*__*D*) is above a certain threshold, the pathway pair *p*_*i*_will be regarded as the pathway profiles for associating a disease with the drugs treating the disease.

Given a new drug, the score of the drug used for treating the disease D can be defined as below:

$$P\left(C_{i}\_D\right)=1\sum_{m=1}^{3}\alpha_{m}\prod \left(1-P\left(p_{m}\_D\right)\right)$$ (2)

where *m* represents one of the three types of pathway profiles, i.e. common, cross-talking and interacting pathway(s), *P*(*p*_*m*__*D*) is the score of the *m*th type of pathway profiles associated with disease *D,* and *α*_*m*_ is the weight for the *m*th type of pathway profiles. To determine the weights for the three types of pathway profiles, the 5-fold cross validation was employed and the AUC was used to choose the proper values. As a result, the weights in Equation (2) were determined as: *α*_1_ = 0.5, *α*_2_ = 0.3, *α*_3_ = 0.2, where the best results were obtained.

#### Predicting new indications of compounds based on the nearest neighbor profile

It has been found that similar drugs tend to have similar mechanism and can be used to treat similar diseases, and *vice versa* [24]. Therefore, given a new drug, the new indications of the drug can be predicted based on its similarity with other drugs. Here, the nearest neighbor profile approach proposed by Yamanishi *et al* [26], *i.e.* nearest profile based on chemical similarity and nearest profile based on disease similarity we named as NP__C_ and NP__D_, was adopted to predict whether a new drug could be used for a certain disease. The chemical similarity between compounds is calculated based on their fingerprints by using the Single Linkage algorithm [31]. The disease similarities are defined in [23], where the similarity was calculated based on disease descriptions from the OMIM database [60].

#### Predicting new indications of compounds based on an ensemble method

The three independent methods mentioned above, i.e. PIPP, NP__C_ and NP__D_, showed different performance on different datasets. Here, we further proposed an ensemble approach named PINA to predict the compound-disease associations by integrating the pathway profile, chemical similarity and disease similarity. In particular, a weight was set for each method based on its performance on a benchmark dataset, and the ensemble learner was constructed as follows:

$$P\left(C\_D\right)=\sum_{i=1}^{n}W_{i}\cdot M_{i}$$ (3)

where *w*_*i*_ is the weight for each method, and *M*_*i*_ is the output of the *i*th method. Here, the weight for each method is set to the AUC (area under the curve) score of a receiver operating characteristic (ROC) curve. For a given compound, we can use the ensemble approach to predict whether the drug can be used for the disease.

#### Predicting new indications of LDW

To evaluate the performance of our proposed approach, the PINA method was applied to infer the therapeutic indications of TCM and investigate the curative effect between TCM and its individual components. To this end, we chose the LDW as a case study since its chemical constituents and indications were well known. Subsequently, we proposed PINA to the 156 chemical constituents to predict compound-disease associations, where a score was calculated based on Equation (3) as the confidence score of the prediction. To determine the threshold above which a prediction is regarded as positive, the 5-fold cross-validation was employed on the known drug-disease associations, i.e. training set. Especially, the threshold that can lead to the highest F1 score was chosen, where the F1 score can evaluate the overall performance of the learner and is a tradeoff between Precision and Recall. Here, the threshold of 0.6 that can lead to the highest F1 score in the cross-validation was chosen. Then we defined an efficacy score for LDW to a certain disease by considering the synergistic effect of all compounds based on Bayesian models. The efficacy score could be described as follows:

$$P\left(LWDH\_D\right)=1-\prod_{C_{i}\in LWDHW}\left(1-P\left(C_{i}\_D\right)\right)$$ (4)

where *C*_*i*_ is the component of LDW. *P*(*C*_*i*__*D*) is the association score between compound *C*_*i*_ and disease *D* calculated with Equation (3).

## SUPPLEMENTARY MATERIALS FIGURES AND TABLES










