# Supplementary material for: Predicting new indications of compounds with a network pharmacology approach: Liuwei Dihuang Wan as a case study
Source: Oncotarget. 2017 Sep 30;8(55):93957–68. doi: 10.18632/oncotarget.21398 (PMC5706847; doi:10.18632/oncotarget.21398)
Supplement: Supplementary file 1 [file oncotarget-08-93957-s001.pdf]

Predicting new indications of compounds with a network pharmacology approach: Liuwei Dihuang Wan as a case study

SUPPLEMENTARY MATERIALS

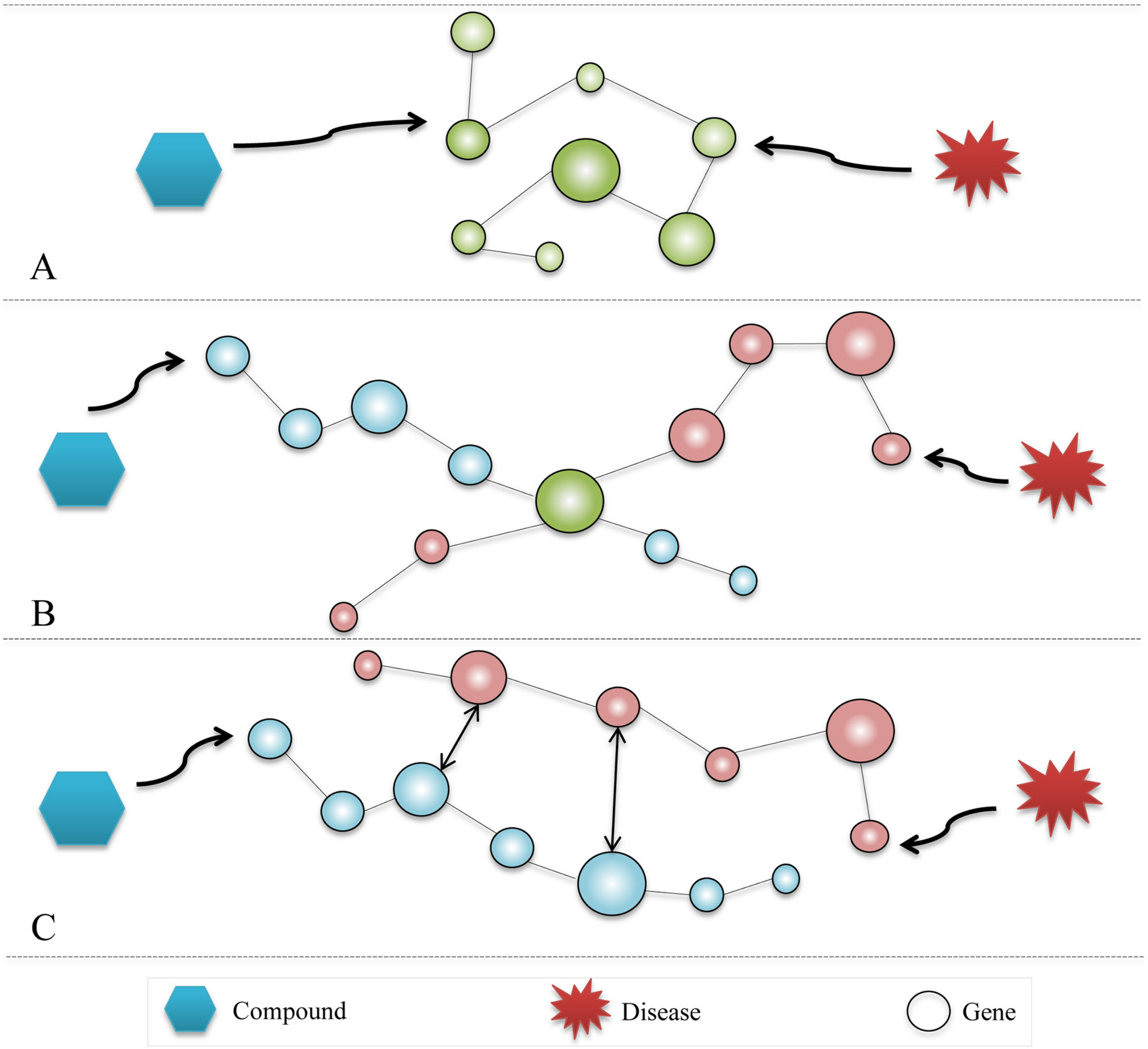

Supplementary Figure 1: Three types of linking pathways. (A) Common pathways (the two pathways are the same one); **(B)** Cross-talking pathways (the two pathways share at least one gene); **(C)** Interacting pathways (there are protein interactions between the two pathways).

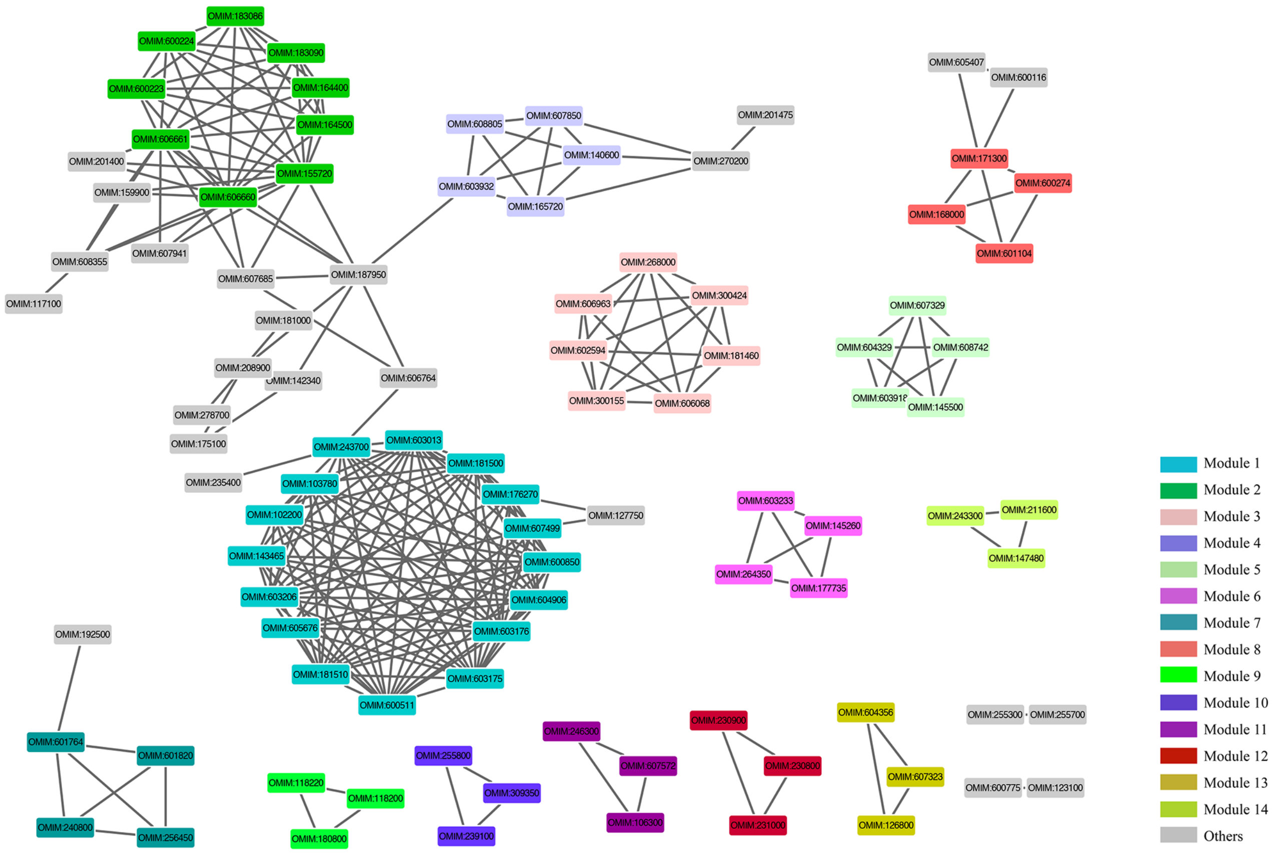

Supplementary Figure 2: Disease-disease associated network. Two diseases were linked if they shared at least one linking pathway where the color of disease were marked based on different modules calculated by MCODE.

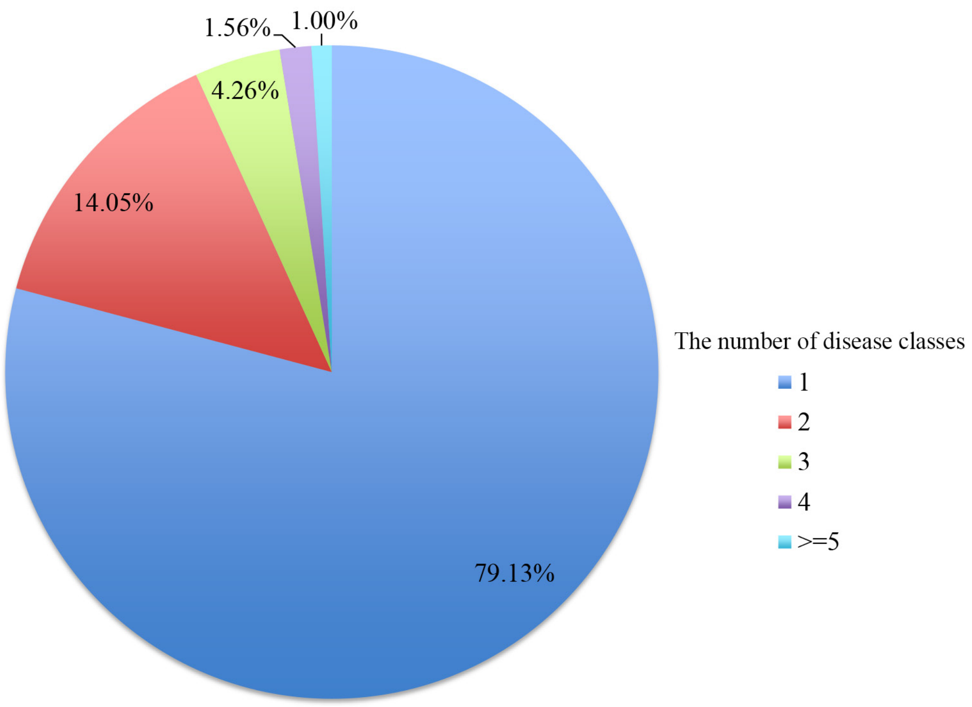

Supplementary Figure 3: The distribution of linking pathways that associated with disease classes. The color represents the number of disease classes.

Supplementary Table 1: 156 compounds of LDW with target information. The first column represents the PubChem CID of the compound and the second column denotes the corresponding target information.

See Supplementary File 1

Supplementary Table 2: The detailed information about the modules detected in disease network with MCODE.

See Supplementary File 2

Supplementary Table 3: The predicted diseases associated with LDW. Green: the well accepted diseases associated with LDW; Red: the disease identified in Li *et al* paper which was a paper specialized with LDW; Blue: the diseases identified with LDW based on text mining; Black: Not available associations with LDW.

See Supplementary File 3

Supplementary Table 4: The novel indications of all the compounds with corresponding scores.

See Supplementary File 4
